# Personality as a predictor of symptomatic change in a residential treatment setting for anorexia nervosa and bulimia nervosa

**DOI:** 10.1007/s40519-020-01023-1

**Published:** 2020-10-13

**Authors:** Laura Muzi, Laura Tieghi, Michele Angelo Rugo, Vittorio Lingiardi

**Affiliations:** 1 Department of Dynamic and Clinical Psychology, Faculty of Medicine and Psychology, Sapienza University of Rome, Via degli Apuli, 1, 00185 Rome, Italy; 2 Eating Disorder Clinic “Residenza Gruber”, Bologna, Italy

**Keywords:** Personality disorders, Eating disorders, Therapy outcome, Clinical significance, Residential treatment

## Abstract

**Purpose:**

Although personality has been widely researched in patients with anorexia nervosa (AN) and bulimia nervosa (BN), the nature of this relationship has not yet been clearly articulated. The *pathoplasty model* theorizes that personality might shape symptomatic presentation and thus affect therapeutic outcomes, but more research is needed. The present study aimed at investigating the predictive value of a broad spectrum of personality traits in determining AN and BN treatment outcomes, considering both the statistical and clinical significance of the therapeutic change.

**Methods:**

Eighty-four female patients with AN and BN treated in a residential program were evaluated at treatment onset using the Shedler-Westen Assessment Procedure-200—a clinician-rated measure of personality disorders and healthy personality functioning. At both intake and discharge, patients completed the Eating Disorder Inventory-3 to assess eating symptoms and the Outcome Questionnaire-45.2 to evaluate overall impairment.

**Results:**

Considering overall ED symptomatic change, multiple regression analyses showed that, even when controlling for baseline symptoms and DSM-5 categories, schizoid (*B* = 0.41, *p* ≤ 0.01), avoidant (*B* = 0.31, *p* ≤ 0.05), and paranoid (*B* = 0.25, *p* ≤ 0.05) personality features predicted worse therapeutic outcomes. Similar results were found when applying the clinical significance approach, with the emotionally dysregulated factor as an additional negative predictor of significant/reliable change (*B* =  − 0.09; *p* < 0.01). Healthy personality functioning predicted better therapeutic outcomes (*B* =  − 0.34, *p* ≤ 0.001).

**Conclusions:**

Pathoplastic models and personality-based research in this clinical population have the potential to inform effective treatment strategies by targeting relevant individual factors.

**Level of evidence:**

Level III, longitudinal cohort study.

## Introduction

Eating disorders (EDs), such as anorexia nervosa (AN) and bulimia nervosa (BN), are complex psychiatric disorders associated with chronicity, decreased psychosocial functioning, a high risk of mortality, and medical complications [1, 2]. Furthermore, failure, relapse, and treatment drop-out rates are particularly high [3]. While psychotherapeutic approaches have demonstrated overall effectiveness in reducing symptomatic impairment [4], most practice guidelines [e.g., 5] agree that a comprehensive evaluation of patients’ individual characteristics should be used to predict and understand patients’ responses to different therapeutic options. Nonetheless, research on reliable outcome predictors at the individual patient level has yielded limited or conflicting results [6]. To date, studies have generally failed to show that ED-specific characteristics, such as those included in the current edition of the *Diagnostic and Statistical Manual of Mental Disorders* (DSM-5) [7], account for patient differences in treatment response [8]. Conversely, some systematic reviews have found that certain baseline transdiagnostic factors, such as better interpersonal functioning, relate to better outcomes [9]. One promising transdiagnostic approach to determining reliable predictors of treatment outcomes considers personality as a relevant “context” [10] in which ED symptoms may serve different functions and provide alternative meanings. In line with early psychodynamic theorists, who interpreted patients’ discrete symptoms through the lens of their characterological context and subjective experiences, the current *pathoplasty model* posits that personality may impact the expression of several forms of psychopathology after onset [11]. Specifically, it may influence the severity or pattern of symptomatology as well as the course of the illness and therapeutic change [12, 13]. Accordingly, previous investigations have shown that personality can predict responses to treatment for depression [14], substance use disorders [15], somatic disorders [16], pathological gambling [17], and other mental disorders [10].

In the ED empirical literature, many studies have addressed the pathoplastic hypothesis, albeit with mixed results [11]. Evidence has shown that personality can explain meaningful variance in the onset, course, maintenance, symptomatic presentation, and recovery rates of ED patients [18–20], as well as their high attrition and low compliance to therapeutic interventions [21]. Impaired personality functioning has also been found to predict differences in the number of previous hospitalizations, treatment length, and overall ED symptoms at the termination of outpatient treatment, over and above the presence of specific ED symptoms [22–24]. In more intensive care settings, personality patterns have been found to be associated with drop-out rates [25]. However, other studies have found no associations between personality functioning/disorders and reliable indices of therapy outcome: for instance, individual DSM-based personality disorders have not been found to predict changes in ED symptoms over time [26], or differential treatment responses in AN and/or BN samples with or without comorbid personality disturbances [27]. Thus, to date, very few reliable conclusions can be drawn regarding the pathoplastic hypothesis of the impact of specific personality disorder features on therapeutic change in this clinical population.

Some limitations of the aforementioned investigations should be noted. First, their examination of personality traits within the boundaries of main ED categories may be limited due to the substantial overlap across the range of ED diagnoses, relating to their common “diagnostic cross-over” and temporal instability [28]. Second, the studies investigated treatment responses primarily through traditional significance testing, which does not provide information regarding within-patient variation or the clinical relevance of therapeutic gains at treatment termination. Conversely, several authors pointed to an increased need to operationalize therapy outcomes also at the individual level, considering both the statistical and the clinical significance of symptomatic change, as well as related predictors [29, 30]. Clinical significance methods, such as the well-known approach proposed by Jacobson and Truax [31], typically classify treatment response at the individual level (i.e., calculating the proportion of patients who made a significant and reliable change, and those who did not) combined with an examination of whether individual patients moved from the dysfunctional to the functional population. Third, few studies employed a multi-informant and dimensional assessment of ED symptoms and personality psychopathology [32], and most applied the five-factor model of personality, which was not designed to specifically assess personality disorder features. Finally, very few of the investigations considered specific personality constellations aside from borderline and obsessive–compulsive personality patterns; however, some authors argued that other personality disorders and traits, such as avoidant personality [33] or suspiciousness and paranoid features [34], may have interacted with other patient variables in predicting ED symptoms over the long term.

The current study attempted to fill this gap in the literature by applying an empirically grounded, clinician-report, and Q-sort procedure—the Shedler-Westen Assessment Procedure-200 (SWAP-200) [35, 36]—to assess a wide range of personality disorder features in AN and BN patients and the predictive value of these features in determining therapy outcomes, considering both the statistical and the clinical significance of the therapeutic change. The SWAP-200 has shown promising effectiveness in identifying personality constellations in AN and BN patients in outpatient treatment [22, 23], as well as their link to identity disturbances and affective functioning [37] and patients’ global psychological adjustment at the end of treatment, after controlling for ED symptoms [38, 39]. Furthermore, its approach is intimately tied to a psychodynamic conceptualization of personality [see 10], which includes three primary functional domains: (a) motivation, desires, values, and wishes, as well as potential conflicts among these; (b) affective, cognitive, and self-regulatory psychological resources; and (c) interpersonal functioning (i.e., how the individual experiences the self and others, as well as the affective quality and complexity of his/her internal representations). Thus, the SWAP-200 has the potential to enhance empirical findings on the pathoplasty model by allowing clinicians to provide in-depth descriptions of both patients’ observable symptoms and their underlying psychological processes, which give rise to ED-related characteristics [40]. Thus, the aims of the study were as follows: (1) taking into account the sample as a whole (i.e., the “group level”), to examine the predictive value of personality disorder features and relevant baseline clinical variables on therapeutic outcomes, considering the severity of ED symptomatic impairment at treatment discharge while controlling for DSM-5 ED diagnoses and baseline ED symptoms; and (2) applying the clinical significance method to systematically assess patients’ variability in treatment outcome (i.e., the “individual level”), to explore whether personality disorder features predicted patients’ differential responses to treatment and their shift to the functional population.

## Method

### Participants

Participants were patients who had been consecutively admitted to a specialized and psychodynamic-oriented residential treatment center for ED in Bologna (Italy) between December 2017 and November 2019. The inclusion criteria were: (a) at least 18 years of age; (b) a diagnosis of DSM-5 anorexia nervosa (AN) or bulimia nervosa (BN), established at intake by the consensus of a licensed staff psychiatrist and a clinical psychologist and based on the Structured Clinical Interview for DSM-5 (SCID-5-CV) [41]; and (c) no organic syndrome, psychotic disorder, or syndrome with psychotic symptoms.

Of the 142 patients who were invited to take part in the study, 26 declined to participate (81.7% response rate). Thus, an initial sample of *N* = 116 met the criteria. Eighteen patients (15.5%) were excluded due to premature discharge or dropout. Patients were reported to have prematurely left the residential program due to scarce personal motivation (*N* = 8, 44.4%), transfer to a psychiatric unit (*N* = 5, 27.7%) or hospital setting (*N* = 4, 22.2%), or a high risk of suicide (*N* = 1, 5.5%). Twelve patients (10.3%) were unable to complete the assessments due to poor medical health or a failure to provide data at intake and/or discharge. Finally, two patients (1.7%) were male and thus not considered in the analyses. Out of the final study sample of *N* = 84, 38 (45.2%) were diagnosed with AN-restricting subtype (AN-R), with an average baseline BMI of 15.16 kg/m^2^, while 14 (16.7%) fulfilled the diagnostic criteria for AN-purging subtype (AN-P), with an average BMI of 16.82 kg/m^2^; the remaining 32 patients (38.1%) were diagnosed with bulimia nervosa (BN), with an average baseline BMI of 22.85 kg/m^2^. Their mean age was 24.19 (SD = 8.38), and all were White/Caucasian. Years of education ranged from 10 to 18 (*M* = 12.89, SD = 3.82). Most patients were single or separated (*N* = 79, 94.1%) and had no previous instance of hospitalization in a psychiatric unit (*N* = 71, 61.2%). At treatment intake, patients reported an average number of 13.7 (SD = 5.25) dietary restrictions per week, 5.51 (SD = 2.89) compensatory behaviors per week, and 3.95 (SD = 2.67) binge episodes per week. Their mean age of ED onset was 16.07 (SD = 3.68). The majority of patients (*N* = 56, 66.6%) also showed at least one comorbid personality disorder, as assessed by the SCID-5-CV. Specifically, 19 patients fulfilled the diagnostic criteria for borderline personality disorder (22.6%), 17 for obsessive–compulsive personality disorder (20.2%), and 10 for avoidant personality disorder (11.9%). Furthermore, 23 patients received a concurrent diagnosis of major depressive disorder (27.4%), 16 (19.1%) an anxiety disorder, and 14 (16.6%) obsessive–compulsive disorder. Other comorbid personality disorders or clinical syndromes were present in less than 5% of patients. Table 1 presents the descriptive characteristics of the study sample with respect to baseline personality features. AN patients showed more schizoid, avoidant, and obsessive personality features, whereas BN patients showed more antisocial, borderline, histrionic, and dependent personality characteristics.

**Table 1:** Baseline personality characteristics of the study sample

Variable	Cronbach’s α	AN patients (*N* = 52)	BN patients (*N* = 32)	Test statistic	*p*	Total sample (*N* = 84)
		M (SD)	M (SD)			*M (SD)*
SWAP-200^a^ personality disorder scales						
Paranoid	0.78	42.69 (5.90)	40.52 (6.40)	1.67	0.22	41.62 (6.12)
Schizoid	0.82	52.70 (8.16)	45.52 (8.36)	14.73	** < 0.001**	49.99 (8.89)
Schizotypal	0.80	49.77 (7.78)	47.02 (9.50)	2.09	0.15	48.72 (8.53)
Antisocial	0.75	41.55 (3.78)	44.08 (5.04)	6.82	**0.01**	42.51 (4.45)
Borderline	0.79	44.78 (7.26)	53.83 (5.76)	35.70	** < 0.001**	48.23 (7.85)
Histrionic	0.74	44.72 (6.33)	51.65 (6.53)	23.84	** < 0.001**	47.98 (7.24)
Narcissistic	0.72	42.86 (5.69)	45.43 (6.27)	3.71	0.06	43.84 (6.01)
Avoidant	0.81	51.88 (7.75)	43.93 (8.21)	19.85	** < 0.001**	48.85 (8.78)
Dependent	0.77	50.39 (6.71)	48.02 (8.18)	2.08	0.15	49.48 (7.35)
Obsessive	0.80	53.44 (7.02)	44.11 (6.44)	37.11	** < 0.001**	49.88 (8.16)
SWAP-200^a^ Q-factors						
Antisocial-Psychopathic	0.72	41.87 (3.58)	43.41 (4.98)	2.70	0.10	42.46 (4.21)
Schizoid	0.84	52.31 (7.77)	46.27 (8.29)	11.34	**0.001**	50.01 (7.45)
Paranoid	0.77	43.40 (6.45)	41.66 (6.49)	1.44	0.23	42.74 (6.48)
Obsessive	0.80	49.93 (7.60)	46.62 (5.95)	4.40	**0.04**	48.67 (7.17)
Histrionic	0.71	43.84 (7.56)	50.09 (9.57)	9.93	**0.002**	46.22 (8.28)
Narcissistic	0.74	49.08 (6.93)	47.32 (6.58)	1.32	0.26	48.41 (6.81)
DS: avoidant	0.83	54.29 (7.45)	46.53 (7.31)	21.73	** < 0.001**	51.33 (8.27)
DS: dependent-masochistic	0.73	48.41 (7.19)	52.31 (6.98)	5.95	**0.02**	49.90 (7.32)
DS: depressive-HF	0.82	50.86 (6.38)	50.55 (6.54)	0.05	0.83	50.74 (6.40)
DS: emotionally dysregulated	0.76	49.63 (7.96)	51.26 (6.91)	1.19	0.28	50.25 (7.58)
DS: hostile-externalizing	0.71	38.14 (7.37)	41.40 (6.14)	2.28	0.10	39.54 (6.68)
Healthy personality functioning	0.85	50.88 (6.65)	51.01 (6.40)	0.01	0.93	50.93 (6.55)

Significant *p* values were reported in bold

*DS* dysphoric Q-factor, *HF* high functioning

^a^Shedler-Westen Assessment Procedure-200 [35, 36]

Seven therapists (all female) participated in the study. Their mean age was 42.7 (SD = 3.76; range = 37–49). The main self-reported clinical orientations were psychodynamic (*N* = 6, 85.7%) and cognitive behavioral (*N* = 1, 14.3%); all were clinical psychologists. The average length of clinical psychotherapy practice was 10.1 years (SD = 3.07; range = 7–15) and the average time spent per week practicing psychotherapy was 22.7 h (SD = 5.14; range = 15–35).

### Residential treatment program

Once patients were admitted, they participated in a full-time non–hospital-based and multidisciplinary residential treatment program with a predominantly psychodynamic approach [42]; see also 29]. Average treatment length was 5.31 months (SD = 2.06, range = 3–12.3). The program was based on a team approach with a patient-tailored perspective. Thus, a multidisciplinary team involving all medical professionals (i.e., psychiatrists, psychologists, social workers, nutritionists, nurses) met on a weekly basis to discuss individual cases within a psychodynamic theoretical framework. They offered patients 24-h supervision to interrupt repetitive and pervasive ED behaviors. Each patient received individual psychotherapy once or twice a week on the basis of a comprehensive examination of their social, psychological, and nutritional status. Other program activities included nutritional rehabilitation and counseling, meal support, interventions focused on affective and emotional experiences, skills training, recreational and art therapy, and social cooking.

### Measures

*Structured Clinical Interview for DSM-5, Clinical Version (SCID-5-CV).* The SCID-5-CV [41] is a semi-structured interview that was designed to categorically assess psychopathology according to the DSM-5. It is typically administered by a clinician who is familiar with the DSM-5 diagnostic criteria. Interview questions are provided alongside each DSM-5 criterion to assist users in rating each criterion as either present or absent. The previous version of the interview (SCID-IV) was found to show good interrater and test–retest reliability [43].

*Clinical Diagnostic Interview (CDI).* The CDI [44] is a 2-h systematic clinical interview comprised of 15 broad questions. Although the CDI includes direct questions where appropriate, it does not rely exclusively on the patient’s description of their presenting symptoms and personality characteristics. Rather, it asks them to tell narratives about their life and relationships, which inform the clinician’s systematic clinical judgment about the patient’s characteristic ways of thinking, feeling, regulating emotions, and self/other representations (e.g., “Can you describe a specific encounter with your mother? Something that stands out. It can be an episode that’s typical of your relationship, really meaningful, really good, really bad—whatever comes to mind.”). Thus, the interview aims at collecting the necessary information for a complete assessment of the patient’s personality, as retrieved through the SWAP-200 (see below). The interview protocol has been shown to have high reliability and validity with patient data across several domains of functioning [45].

*Shedler-Westen Assessment Procedure–200 (SWAP-200).* The SWAP-200 [35, 36] is a psychometric procedure that was designed to provide a comprehensive assessment of personality functioning. With the aid of a computerized program, the instrument utilizes a Q-sort method, which requires the rater to sort 200 items into eight categories, ranging from 0 (*not descriptive*) to 7 (*most descriptive*) of the individual, to ensure a fixed distribution. SWAP-200 items are written in a straightforward, experience-near language (e.g., “Tends to be passive and unassertive,” “Has an exaggerated sense of self-importance”); this is especially true of items that require clinical judgment about internal psychological processes (e.g., “Tends to see own unacceptable feelings or impulses in other people instead of in him/herself”). In line with the growing consensus on the limitations of categorical conceptualizations of personality [46], in the present study, we used only dimensional scores. The SWAP-200 scoring algorithms generate: (a) a personality diagnosis based on the matching of the patient assessment with 10 personality disorder scales, which are prototypical descriptions of DSM-5 personality disorders (PD scales); and (b) a personality diagnosis based on the matching of the patient’s SWAP description with 11 personality styles derived from a Q-analysis. Q-analysis uses the same algorithms as factor analysis, but it creates groupings of people, not variables. The resulting groups (Q-factors) provide an alternative set of personality syndromes that have been empirically identified and resemble personality patterns found in clinical practice. Several aspects of the SWAP-200 Q-factors are worthy of note, as they differ from DSM-5 diagnostic categories. First, the Q-analysis showed that many patients were classified as belonging in the dysphoric Q-factor; this describes individuals with a tendency to feel distressed in multiple ways and experience feelings of inadequacy, shame, guilt, depression, and fear of rejection or abandonment, but in different activating conditions. Thus, a second Q-analysis identified five subgroups within the dysphoric Q-factor: avoidant; dependent-masochistic; depressive–high functioning; emotionally dysregulated; and hostile-externalizing. Second, a single schizoid Q-factor emerged that included many patients currently diagnosed as schizoid and schizotypal. Third, patients currently diagnosed as borderline tended to fall into either the histrionic or the dysphoric: emotionally dysregulated and hostile-externalizing Q-factors. Finally, the obsessive Q-factor appeared to be substantially less disturbed than implied by the current DSM-5 conceptualization [for a wider description, see 36]. In addition to producing PD scale and Q-factor scores, the SWAP-200 also provides a “healthy personality functioning” score. The measure has been shown to have excellent test–retest reliability, as well as good interrater, discriminant, and convergent validities [47, 48]. Cronbach’s alphas for each SWAP-200 PD scale and Q-factor in the present study are reported in Table 1.

*Outcome Questionnaire-45.2 (OQ-45.2).* The OQ-45.2 [49] is a 45-item self-report instrument that was designed to measure important areas of functioning and symptomatic impairment (i.e., symptoms, interpersonal problems, social roles) that are of central interest to mental health (e.g., “I feel something is wrong with my mind,” “I feel unhappy in my marriage/significant relationship,” “I feel stressed at work/school”). Each item is rated on a 5-point Likert scale ranging from 0 (*never*) to 4 (*almost always*). The sum of item scores (after reverse coding selected items) provides a total score, which was used in the present study. In prior studies, the measure has been found to demonstrate good internal consistency and test–retest reliability [50]. In the present study, Cronbach’s alpha for the OQ-45.2 total score was 0.91.

*Eating Disorder Inventory-3 (EDI-3).* The EDI-3 [51] is a self-report questionnaire that is widely used in both research and clinical settings to assess the core components of eating psychopathology. It consists of 91 items organized into 12 primary scales, consisting of 3 ED-specific scales and 9 general psychological scales that are highly relevant to EDs (e.g., “I think my stomach is too big,” “When I am upset, I worry that I will start eating”). It also yields six composite scores: one that is ED-specific and five that are general integrative psychological constructs. In the present study, we considered the Global Psychological Maladjustment composite score as an index of overall ED symptomatic impairment. The EDI-3 has been found to yield adequate convergent and discriminant validity [52]. In the present study, Cronbach’s alphas for EDI-3 scores ranged from 0.72 to 0.93.

### Procedure

Potential participants had been previously referred to the residential treatment program in Bologna, Italy, for the treatment of an ED by a family doctor or other clinicians from the National Health Service. At treatment intake, patients were asked to participate in a research protocol on psychological assessment and their experiences of therapy. During the first week of treatment, all patients who agreed to participate were evaluated with the SCID-5-CV by a licensed staff psychiatrist and a clinical psychologist, to ensure fulfillment of the inclusion criteria and assess comorbid diagnoses. Height and body weight were also measured to calculate BMI at treatment intake. Moreover, during the first and last week of treatment, all patients completed self-report measures to assess ED-specific symptoms and overall psychopathology. To minimize the effect of acute starvation and acute ED symptoms on personality, the CDI and SWAP-200 assessment were administered within the first 2 weeks after admission (instead of in the first week, as with the other assessments). Psychotherapists were trained to use the SWAP-200 with the CDI in a 16-h workshop led by the first and last authors of this article. The last author, in turn, was trained with the SWAP and CDI by Drew Westen and Jonathan Shedler. All study subjects participated voluntarily and provided written informed consent prior to the assessments, following the review and approval of the study protocol by the local research ethics committee.

### Statistical analyses

*Correlations and hierarchical regression analyses.* The first outcome index, at the group level, was the self-reported EDI-3 overall score at termination. Univariate correlations between SWAP-200 scales, socio-demographic characteristics, and clinical variables on the one hand, and symptom score on the other, were calculated. Variables and SWAP-200 scales showing significant associations were entered into separate hierarchical multiple regressions. Specifically, we tested separate models for baseline variables, SWAP-200 PD and Q-factor scores, and the SWAP-200 healthy personality functioning score. All multiple regressions were estimated in three steps. In the first step (i.e., block), EDI-3 score at treatment intake was included to create a residual gain score and thereby control for baseline symptoms. DSM-5 diagnostic categories (i.e., AN or BN) were added in the second step. Finally, variables and SWAP-200 scales were entered in the third step. Change in R^2^ was used to measure the significance of each step. The F test, which is referred to as F-change, was used to test whether R^2^ improvement was statistically significant. All continuous variables were grand mean centered to reduce collinearity.

*Clinical significance.* The second outcome index was at the individual level, with the clinical significance of therapeutic change determined according to the criteria proposed by Jacobson and Truax [31]. To determine whether a patient’s change was reliable or the result of measurement error or chance, a reliable change index (RCI) was calculated by subtracting the post-treatment score from the pre-treatment score and dividing the resulting figure by the standard error of the difference between the test scores. Patient change was considered reliable when it exceeded the measurement error at a 0.05 level of confidence. In the second step, a cut-off point (i.e., “criterion c”) was determined to assess whether a patient outcome score fell within the functional or dysfunctional population range. In the Italian population, the cut-off score was estimated as a total OQ-45.2 score of 66 [53]. Thus, the sample was classified into four outcome groups: (a) *clinically significant improvement*, with a pre–post decrease in OQ-45.2 total score of more than 14 points and a cut-off score in the functional range; (b) *reliable improvement*, with a significant pre–post decrease in OQ-45.2 total score but a cut-off score in the dysfunctional range; (c) *no change*, with no reliable change and a cut-off score in the dysfunctional range; and (d) *reliable deterioration*, with a pre–post increase in OQ-45.2 total score of more than 14 points and a cut-off score in the dysfunctional range [54].

*Statistical group comparisons and binary logistic regression analyses.* For the purpose of this study, patients were divided into two groups according to clinical significance methodology: those showing clinically significant/reliable therapeutic change and those showing no such change (i.e., unchanged and deteriorated patients). To explore the differences between these outcome groups, we first computed separate univariate ANOVAs. Second, we ran a binary logistic regression model, with all variables showing significant differences between patient groups in previous analyses and no multicollinearity entered as predictors. We set the significance level of the Wald chi-square for an effect to remain in the model to 0.05. The results were expressed as odds ratios with corresponding 95% confidence intervals (CIs). Nagelkerke’s *R*^2^ was chosen as the estimate of explained variance.

All analyses were conducted using SPSS 25 for Windows. As previously mentioned, any patient missing ED symptoms and overall symptomatic impairment assessments at treatment intake and/or discharge were not included in the analyses. We handled missing values for individual outcome measures by replacing them with the mean score of the specific item. Due to the software’s fixed distribution requirement, no SWAP-200 data were missing.

## Results

### Personality and clinical variables predicting symptomatic change at the group level

As shown in Table 2, with respect to clinical variables, higher EDI-3 symptom scores at discharge were positively associated with the number of dietary restrictions per week and negatively related to treatment length. Considering the SWAP-200 PD scales, our results showed positive associations between higher ED symptomatic impairment at discharge and paranoid, schizoid, schizotypal, and avoidant PD scales, as well as schizoid, paranoid, histrionic, dysphoric: avoidant, and dysphoric: emotionally dysregulated Q-factors. Conversely, higher healthy personality functioning scores were related to lower ED symptom scores, as well as obsessive and dysphoric: depressive–high functioning Q-factors. Of note, the borderline and obsessive–compulsive PD scales were not significantly associated with the symptomatic condition at discharge.

**Table 2:** Associations between baseline variables, personality characteristics, and ED symptoms at discharge (*N* = 84)

	EDI-3^a^ overall symptomatic score	*p*
Socio-demographic variables		
Age (years)	− 0.142	0.09
Education (years)	− 0.009	0.39
Clinical variables		
Treatment length	− 0.237	**0.01**
BMI (baseline)	− 0.163	0.07
Age of ED onset	− 0.156	0.08
Dietary restrictions/week	0.332	**0.001**
Compensatory behaviors/week	0.096	0.19
Binge eating episodes/week	0.002	0.49
SWAP-200^b^ PD scales		
Paranoid	0.268	**0.007**
Schizoid	0.357	** < 0.001**
Schizotypal	0.395	** < 0.001**
Antisocial	0.066	0.27
Borderline	0.058	0.30
Histrionic	0.009	0.40
Narcissistic	0.139	0.10
Avoidant	0.255	**0.01**
Dependent	0.004	0.48
Obsessive	0.099	0.18
SWAP-200^b^ Q-factors		
Antisocial-Psychopathic	0.037	0.37
Schizoid	0.336	**0.001**
Paranoid	0.252	**0.01**
Obsessive	− 0.210	**0.03**
Histrionic	0.256	**0.02**
Narcissistic	0.051	0.32
DS: avoidant	0.214	**0.02**
DS: dependent-masochistic	0.112	0.15
DS: depressive-HF	− 0.253	**0.01**
DS: emotionally dysregulated	0.260	**0.008**
DS: hostile-externalizing	0.034	0.38
Healthy personality functioning	− 0.430	** < 0.001**

Significant *p* values were reported in bold

*DS* dysphoric Q-factor, *HF* high functioning

^a^Eating Disorder Inventory-3 [51]; ^b^Shedler-Westen Assessment Procedure-200 [35, 36]

As a next step, we entered significant variables into hierarchical multiple regressions. Given the moderate sample size, to preserve statistical power, only variables that showed a significant association in the previous analyses were retained. Table 3 shows that even when controlling for baseline ED symptoms and DSM-5 ED categories, a greater number of dietary restrictions emerged as a significant predictor of higher ED symptoms at discharge. With respect to personality characteristics, the schizoid, avoidant, and paranoid PD scales and Q-factors emerged as significant predictors of higher ED symptomatic impairment at discharge. Conversely, healthy personality functioning and dysphoric: depressive–high functioning predicted lower ED symptom scores at discharge (see Table 3). Of note, baseline ED symptomatic impairment emerged as a significant predictor in all regression models, whereas DSM-5 ED categories did not affect therapeutic outcomes at a group level.

**Table 3:** Hierarchical multiple regression analyses predicting ED symptoms at discharge (*N* = 84)

Criterion variable: EDI-3 overall score	Step	*R*	*R*^*2*^	Standardized β	F-change (Model)	*p*
Model 1: Clinical variables						
Baseline ED symptoms^a^	1	0.434	0.189	0.434	19.079	< 0.001
DSM-5 ED categories (AN = 0, BN = 1)	2	0.453	0.205	0.129	1.684	0.198
Clinical variables (all predictors)	3	0.600	0.360		9.551	< 0.001
Treatment length				0.140		
Dietary restrictions/week				0.336***		
Model 2: SWAP-200 PD scales						
Baseline ED symptoms	1	0.434	0.189	0.434	19.079	< 0.001
DSM-5 ED categories (AN = 0, BN = 1)	2	0.453	0.205	0.129	1.684	0.198
PD scales (all predictors)	3	0.681	0.421		7.728	< 0.001
Paranoid				0.254*		
Schizoid				0.411**		
Schizotypal				0.198		
Histrionic				0.559		
Avoidant				0.311*		
Model 3: SWAP-200 Q-factors						
Baseline ED symptoms	1	0.434	0.189	0.434	19.079	< 0.001
DSM-5 ED categories (AN = 0, BN = 1)	2	0.453	0.205	0.129	1.684	0.198
Q-factors (all predictors)	3	0.681	0.421		7.728	< 0.001
Schizoid				0.388**		
Paranoid				0.261*		
Obsessive				0.123		
DS^c^: avoidant				0.273*		
DS: depressive-HF				− 0.302*		
DS: emotionally dysregulated				0.158		
Model 4: personality functioning						
Baseline ED symptoms	1	0.434	0.189	0.434	19.079	< 0.001
DSM-5 ED categories (AN = 0, BN = 1)	2	0.453	0.205	0.129	1.684	0.198
SWAP-200^b^ healthy personality functioning	3	0.559	0.312	− 0.338	12.416	0.001

*DS* dysphoric Q-factor, *HF* high functioning

^a^Eating Disorder Inventory-3 [51]; ^b^Shedler-Westen Assessment Procedure-200 [35, 36]

*p ≤ 0.05; **p ≤ 0.01; ***p ≤ 0.001

### Personality and clinical variables predicting clinically significant/reliable symptomatic change at the individual level

**Fig. 1:**
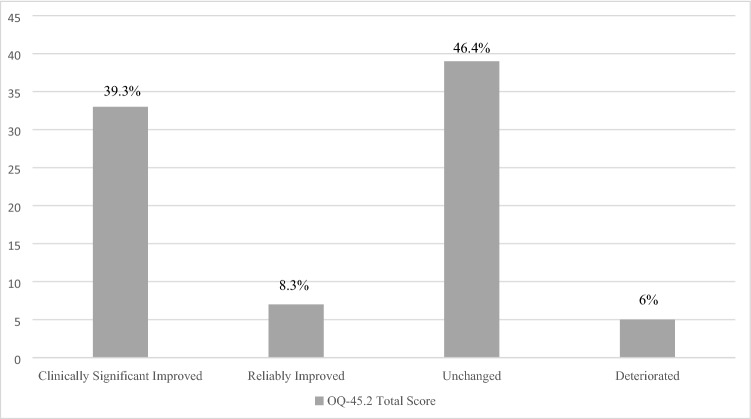
Clinical significance of symptomatic change at discharge (*N* = 84). Notes. Outcome based on the Outcome Questionnaire-45.2 Total score [49]

Figure 1 shows the results pertaining to the clinical significance of the symptomatic change. We found that 39.3% of ED patients fulfilled the criteria for clinically significant improvement, and an additional 8.3% showed reliable symptomatic change, even though they remained within the dysfunctional population. On the other hand, 46.4% showed no significant improvement and 6% deteriorated. As mentioned previously, we grouped together patients who had significantly changed following treatment and those who had not. Table 4 shows all the comparisons between these groups with respect to baseline variables and personality characteristics. Unchanged/deteriorated patients showed an earlier age of ED onset, as well as a greater number of dietary restrictions and compensatory behaviors per week. In terms of personality characteristics, unchanged/deteriorated patients showed more paranoid, schizoid, schizotypal, avoidant, and borderline features with respect to the SWAP-200 PD scales, as well as higher scores for schizoid, paranoid, dysphoric: avoidant, dysphoric: emotionally dysregulated, and dysphoric: hostile-externalizing Q-factors. Conversely, clinically and reliably improved patients were characterized by higher levels of healthy personality functioning, as well as more obsessive and dysphoric: depressive–high functioning scores.

**Table 4:** Comparison of recovered and unchanged groups for baseline variables, SWAP-200 PD Scales, and Q-factors (*N* = 84)

Variable	Clinically significant/ reliable change^b^ (*N* = 40) M (SD)	Unchanged/ deteriorated^b^ (*N* = 44) M (SD)	*F*	*p*	*η*^2^
Socio-demographic variables					
Age (years)	25.16 (6.08)	26.40 (7.55)	1.75	0.18	0.02
Education (years)	13.16 (4.21)	12.52 (3.79)	0.46	0.64	0.007
Clinical variables					
Treatment length	4.58 (2.24)	5.34 (1.88)	0.86	0.34	0.01
BMI (baseline)	18.16 (5.14)	18.59 (5.09)	0.14	0.71	0.002
Age of ED onset	15.15 (3.30)	16.98 (3.80)	4.80	**0.03**	0.06
Dietary restrictions/week	17.48 (7.91)	9.50 (6.40)	25.31	** < 0.001**	0.23
Compensatory behaviors/week	7.86 (5.88)	2.93 (4.19)	15.38	** < 0.001**	0.16
Binge eating episodes/week	4.16 (4.65)	2.82 (4.18)	3.26	0.07	0.03
SWAP-200^a^ PD scales					
Paranoid	38.71 (6.54)	44.26 (4.29)	21.46	** < 0.001**	0.20
Schizoid	45.85 (8.34)	53.75 (7.70)	20.31	** < 0.001**	0.19
Schizotypal	43.62 (6.77)	53.35 (7.26)	40.09	** < 0.001**	0.28
Antisocial	41.70 (4.96)	43.25 (3.83)	2.60	0.11	0.03
Borderline	46.45 (8.06)	49.84 (7.43)	3.86	**0.05**	0.04
Histrionic	47.39 (7.41)	48.21 (7.16)	0.51	0.61	0.008
Narcissistic	43.24 (6.89)	44.39 (5.10)	0.76	0.38	0.01
Avoidant	46.12 (9.06)	51.34 (7.83)	8.03	**0.006**	0.09
Dependent	48.55 (7.65)	50.33 (7.05)	1.24	0.26	0.01
Obsessive	48.55 (9.04)	51.10 (7.15)	2.06	0.15	0.02
SWAP-200^a^ Q-factors					
Antisocial-psychopathic	41.29 (4.80)	43.52 (3.30)	3.13	0.07	0.04
Schizoid	46.11 (7.98)	53.55 (7.30)	19.81	** < 0.001**	0.19
Paranoid	39.53 (6.34)	45.65 (5.14)	23.69	** < 0.001**	0.22
Obsessive	51.02 (6.64)	46.53 (7.03)	8.99	**0.004**	0.10
Histrionic	48.37 (7.96)	44.27 (9.03)	3.48	0.06	0.04
Narcissistic	47.43 (6.86)	49.29 (6.72)	1.57	0.21	0.02
DS^b^: avoidant	49.07 (8.48)	53.40 (7.60)	6.09	**0.01**	0.07
DS: dependent-masochistic	49.67 (6.99)	50.15 (6.60)	0.10	0.74	0.001
DS: depressive-HF	54.04 (5.72)	47.74 (5.48)	26.47	** < 0.001**	0.24
DS: emotionally dysregulated	47.66 (7.83)	52.60 (6.59)	9.83	**0.002**	0.11
DS: hostile-externalizing	37.60 (6.03)	41.30 (5.88)	6.90	**0.01**	0.08
Healthy personality functioning	55.44 (5.59)	46.82 (4.56)	50.32	** < 0.001**	0.30

Significant *p* values were reported in bold

*DS* dysphoric Q-factor, *HF* high functioning

^a^Shedler-Westen Assessment Procedure-200 [35, 36]; ^b^Based on the Outcome Questionnaire-45.2 total score [49]

We entered these variables into binary logistic regression analyses—the results of which are presented in Table 5. Earlier age of ED onset and ED dysfunctional behaviors, such as dietary restrictions and inappropriate compensatory behaviors, were negative predictors of improvement at the individual level. Similar to the previous results, paranoid, schizoid, and avoidant PD scales, as well as the schizoid, paranoid, dysphoric: avoidant, and dysphoric: emotionally dysregulated Q-factors, were negative predictors of therapy outcome. Conversely, healthy personality functioning, obsessive, and dysphoric: depressive–high functioning Q-factors emerged as predictors of clinically significant and reliable symptomatic change.

**Table 5:** Significant predictors of clinically significant and reliable therapeutic change

	B	SE	Wald	Nagelkerke	*p*	EXP(B)	95% CIs for EXP(B)	
				*R*^2^			Lower bound	Upper bound
Model 1: clinical variables				0.73				
Age of ED onset	0.40	0.14	7.60		0.006	1.49	1.12	1.99
Dietary restrictions/week	− 0.41	0.10	15.58		< 0.001	0.67	0.55	0.81
Compensatory behaviors/week	− 0.42	0.11	14.66		< 0.001	0.65	0.52	0.81
Model 2: SWAP-200^a^ PD scales				0.57				
Paranoid PD	− 0.15	0.06	7.16		0.007	0.85	0.76	0.95
Schizoid PD	− 0.21	0.07	9.92		0.002	0.81	0.71	0.92
Avoidant PD	− 0.10	0.03	6.21		0.01	0.93	0.88	0.97
Model 3: SWAP-200^a^ Q-factors				0.68				
Schizoid	− 0.13	0.03	13.55		< 0.001	0.88	0.82	0.94
Paranoid	− 0.18	0.05	14.89		< 0.001	0.83	0.75	0.91
Obsessive	0.09	0.03	7.54		0.006	1.10	1.03	1.18
DS: avoidant	− 0.07	0.03	5.56		0.02	0.93	0.88	0.98
DS: depressive HF	0.20	0.05	15.87		< 0.001	1.23	1.11	1.35
DS: emotionally dysregulated	− 0.09	0.03	8.08		0.004	0.91	0.85	0.97
Model 4: healthy personality functioning	0.36	0.07	21.69	0.56	< 0.001	1.4	1.23	1.68

*DS* dysphoric Q-factor

^a^Shedler-Westen Assessment Procedure-200 [35, 36]. *Outcome* Outcome Questionnaire-45.2 total score [49]

## Discussion

Although a growing body of empirical evidence suggests specific associations between personality and eating pathologies, only rarely have such findings been explicitly presented within a specific conceptual model of the relationship between these variables. Drawing on the theoretical framework of the pathoplasty model [11] and the psychodynamic perspective of the personality as relevant frameworks for understanding ED symptoms [10], the present study aimed at investigating whether personality disorder features, as assessed through a dimensional empirical approach, could predict overall ED symptomatic impairment (at the group level) and the clinical significance of ED symptomatic change (at the individual level) at discharge from a psychodynamic-oriented residential treatment program. To the best of our knowledge, this study was the first to explore the relevance of a broad spectrum of personality traits and styles in determining therapeutic outcomes in patients with AN and BN, also applying clinical significance criteria. The concept of clinical significance, as operationalized through the application of Jacobson and Truax’s [31] formula, can help bridge the gap between empirical research and clinical practice by examining the ways in which individuals respond to treatment, and how their responses might be affected by personality factors.

Overall, our findings supported our hypotheses: even after controlling for baseline ED symptoms and DSM-5 diagnostic categories, patients’ personality disorder features significantly contributed to predicting therapy outcomes at both the group and the individual level. SWAP-200 personality scales characterized by interpersonal distance, detachment, impoverished emotional and cognitive processes, and difficulty drawing meaning from others’ behavior (e.g., schizoid and avoidant PD scales and Q-factors) were strong negative predictors of overall ED symptomatic change and clinically significant/reliable improvement at the individual level. Despite the paucity of studies on the relationship between these personality characteristics and therapy outcomes, several authors have suggested that certain ED symptoms, such as the need for control over body weight and eating patterns, excessive concern over body shape, and feelings of shame or inadequacy, might severely affect intimate relationships and result in interpersonal withdrawal [32, 55]. Furthermore, previous studies that have explored personality subtypes in AN and BN patients with the SWAP-200 have found that patients with an avoidant-insecure style have the worst prognosis [38]. These observations seem particularly relevant to psychodynamic-oriented clinicians, who view the therapeutic relationship as a vehicle or medium for change and tend to place a strong focus on patients’ interpersonal experiences (both early and current) [56]. In residential treatment settings, personality disturbances could lead to severe difficulty fitting into the environment, which is likely to reward strong interpersonal relationships with a treatment team and other patients.

Paranoid personality features also predicted worse therapeutic outcomes at both the group and the individual level. Projective thinking, hostility, high interpersonal distrust and suspiciousness, and a subsequent inability to share thoughts and feelings with other people (including the treatment team) could make it more difficult for patients to establish a positive relationship with their therapists, and thereby interfere with treatment compliance or completion [57, 58]. In line with our findings, Dingemans et al. [34] found that higher levels of interpersonal distrust and suspiciousness were associated with lower chances of a good treatment outcome. Conversely, higher levels of SWAP-200 healthy personality functioning, characterized by mature defense mechanisms, empathy, responsiveness, capacity for relationship and intimacy, nurturance, affective regulation, insight, and reflective capacity [35, 36], were associated with better therapeutic outcomes at both the group and the individual level. While many theoreticians agree that the concepts of *mental health* and *adaptive functioning* are foundational for the definition of mental disorders, descriptive psychiatric taxonomies have not proceeded accordingly. Conversely, therapeutic interventions focused on these “protective factors” related to psychological well-being in AN and BN patients may increase the effectiveness of intervention programs for this clinical population [59].

Our results also showed that, while the unchanged patient group showed more borderline, emotionally dysregulated, and hostile-externalizing personality characteristics, only the emotionally dysregulated Q-factor was a negative predictor of clinically significant/reliable change. While comorbid borderline traits among EDs have been shown to be associated with worse clinical presentations and greater psychiatric disturbance [27, 60], the SWAP-200 results suggest that most patients currently diagnosed with a borderline personality disorder may be better defined by a Q-factor characterized by emotions that spiral out of control, a tendency to become irrational in the face of strong emotions, suicidal and self-harming behaviors, and an inability to self-soothe [36]. This finding echoes studies showing that impulsivity and emotional dysregulation in ED patients are not only associated with higher levels of psychiatric and ED symptoms [61] but also predictive of residual symptoms at the end of treatment and potentially related to unfavorable treatment outcomes [21]. In a residential care setting, such personality characteristics might express themselves as a tendency to break rules and severe difficulty adjusting to the treatment protocol; both of these expressions are likely to worsen therapeutic outcomes.

Similarly, previous studies with the SWAP-200 Q-factors taxonomy have suggested that patients with obsessive–compulsive personality disorder may appear to be significantly less disturbed than the current DSM conceptualization would suggest [36]. In our sample, the obsessive–compulsive PD scale was not found to affect therapeutic change, but the obsessive Q-factor personality predicted clinically significant/reliable change, suggesting that, despite emotional constriction, a tendency to intellectualize, and an excessive concern with rules, these patients showed psychological strengths that may have enhanced their likelihood of a good outcome. Another positive predictor of therapeutic change was the dysphoric: depressive–high functioning Q-factor, characterized by several psychological strengths alongside dysphoric feelings of guilt and shame [62]. Of note, several studies employing the SWAP-200 in AN and BN samples have suggested that a proportion of patients fall within a high-functioning/perfectionistic group characterized by both psychological resources and a tendency towards perfectionism, negative affectivity, and self-criticism; patients in this group tend to show higher overall adaptive functioning, fewer comorbidities, and better global treatment response [22, 23, 37]. This high-functioning profile has also been identified by numerous other research teams [63, 64] using other assessment measures.

In the present study, some clinical variables related to ED pathology and treatment also emerged as significant predictors. Thus, future studies should consider the relationship between such variables and personality disorder features in more depth. A larger number of dietary restrictions per week predicted worse therapeutic outcomes at both the group and the individual level, in line with previous prospective studies showing that restrictive dieting predicted both the onset and the worsening of EDs [65]. Further, in accordance with previous findings on the negative predictive role of recurrent purging behaviors [66], a greater number of inappropriate compensatory behaviors per week negatively affected the likelihood of clinically significant/reliable symptomatic change. Similar results emerged for the earlier age of ED pathology onset, which has previously been found to predict more severe symptomatic impairment and a longer duration of illness [67].

Despite these promising results, our data should be interpreted in light of some relevant limitations. First, the study included a moderate sample size of exclusively White/Caucasian women, which limits the generalizability of the findings to males, minority demographic populations, and populations with baseline EDs other than AN or BN. Second, our results only pertain to patients who were discharged after treatment completion. Thus, future studies should explore which personality variables might affect treatment drop-out or premature discharge in residential treatments for AN and BN patients. Third, data were collected from a single residential treatment center, and the multidisciplinary therapeutic approach did not facilitate a deep understanding of the kind of intervention that is most effective at promoting symptomatic improvement and clinically significant/reliable therapeutic change. Future investigations should explore the impact of personality on therapy outcomes within a more heterogeneous ED patient sample, while also controlling for treatment setting (e.g., outpatient, day treatment program, inpatient, etc.) and therapeutic approach (e.g., psychodynamic, cognitive-behavioral, interpersonal, etc.). However, this limitation may also be a potential strength: as all patients were from the same residential unit and interacted with the same team of professionals, they participated in a shared environment, which facilitated our investigation of the impact of individual factors [24]. An additional limitation is that, after discharge, no assessment of personality, ED symptoms, and/or overall impairment was carried out. In the future, our findings should be supported by longitudinal data collection on personality and baseline clinical variables. Such investigations might also explore changes in personality functioning, as well as the associations between personality factors, the therapeutic relationship (e.g., the therapeutic alliance or therapist effects) [68, 69], and outcomes.

Despite these limitations, the potential strengths of the study include the dimensional approach employed to assess personality, as well as the utilization of both statistical and clinical significance methods to explore therapeutic outcomes. Additionally, the incorporation of clinician measures provided a complementary perspective to the self-report data, which have underpinned most previous findings on personality and ED. Clinicians are experienced observers with longitudinal knowledge of their patients, and previous research has suggested that, when their observations are psychometrically quantified by highly reliable tools, their judgments are valid [70].

The present study also has relevant clinical implications. First and foremost, our findings suggest that if clinicians want to understand and treat ED symptoms effectively, they have to know something about the person who host them [10]. A primary emphasis on observable symptoms may lead clinicians to neglect less overt—and less easily measurable—aspects of patients’ subjective experiences, such as feelings of isolation and loneliness, as well as shame, guilt, and hostility. Accordingly, it is well known that most practitioners begin their psychological evaluations by trying to understand the meaning and function of their AN and BN patients’ difficulties within the larger context of their personality dynamics. In clinical practice, for example, some patients might develop ED symptoms because they are competitive and perfectionistic, while others might use ED symptoms as a way of regulating feelings of being out of control.

Thus, the pathoplasty model and personality-based research in the field of EDs have the potential to inform effective treatment guidelines and strategies by targeting relevant individual factors, such as social withdrawal and avoidance, fear of intimate relationships, interpersonal distrust and suspiciousness, difficulty understanding the mental states of others, feelings of social inferiority, emotional dysregulation, and impulsivity. Moreover, the SWAP-200 healthy personality functioning scale allowed us to derive an overall measure of psychological strengths and resources in different domains of functioning, in line with increasing evidence that both mental health and psychopathology dimensions should be incorporated into the development of effective treatments [71]. Lastly, the high heterogeneity of ED clinical presentations and treatment responses point to the need to consider personality features as potentially stable variables that should be routinely assessed at treatment intake and included in case conceptualizations, to help practitioners develop more patient-tailored avenues for this difficult to treat population. As suggested by some authors [21, 72], a pivotal future direction in ED research and clinical practice will be to shift from a “one-size-fits-all” to a “person(ality)-centered” treatment approach, encouraging practitioners to adapt psychotherapy interventions to suit the specific transdiagnostic characteristics (e.g., personality features) of individual patients, to better meet their needs and enhance their therapeutic outcomes.

### What is already known on this subject?

The relationship between personality and treatment outcomes in AN and BN patients has received considerable empirical testing, but these findings have rarely been presented within a conceptual and clinically meaningful model. To date, studies on the pathoplasty model, which posits that personality traits and disorders may influence therapeutic change, have shown relevant limitations and produced mixed results.

### What does this study add?

Employing a theoretically grounded and multi-informant perspective, the present study showed that under-researched personality disorder features, such as schizoid, avoidant, and paranoid characteristics, predicted worse therapeutic outcomes, considering both the statistical and the clinical significance of therapeutic change. Conversely, personality strengths and resources were found to be positive predictors of symptomatic change. The findings suggest that pathoplasticity may represent a viable model for the integration of personality-based research in the development of effective and patient-tailored treatment strategies for AN and BN patients.

## Data Availability

The datasets generated and/or analyzed in the current study are available from the corresponding author upon reasonable request.
